# Assessing disturbances in surviving primary forests of Europe

**DOI:** 10.1111/cobi.14404

**Published:** 2024-11-19

**Authors:** José I. Barredo, Inés Marí Rivero, Klára Janoušková

**Affiliations:** ^1^ European Commission, Joint Research Centre (JRC) Ispra Italy; ^2^ FINCONS SPA Vimercate Italy; ^3^ ARHS Developments S.A. Luxembourg Luxembourg

**Keywords:** disturbance, disturbance severity, Europe, old‐growth forest, potential primary forest, primary forest, bosque antiguo, bosque primario, bosque primario potencial, Europa, perturbación, severidad de la perturbación

## Abstract

Primary forests are of paramount importance for biodiversity conservation and the provision of ecosystem services. In Europe, these forests are scarce and threatened by human activities. However, a comprehensive assessment of the magnitude of disturbances in these forests is lacking, due in part to their incomplete mapping. We sought to provide a systematic assessment of disturbances in primary forests in Europe based on remotely sensed imagery from 1986 to 2020. We assessed the total area disturbed, rate of area disturbed, and disturbance severity, at the country, biogeographical, and continental level. Maps of potential primary forests were used to mitigate gaps in maps of documented primary forests. We found a widespread and significant increase in primary forest disturbance rates across Europe and heightened disturbance severity in many biogeographical regions. These findings are consistent with current evidence and associate the ongoing decline of primary forests in Europe with human activity in many jurisdictions. Considering the limited extent of primary forests in Europe and the high risk of their further loss, urgent and decisive measures are imperative to ensure the strict protection of remnants of these invaluable forests. This includes the establishment of protected areas around primary forests, expansion of old‐growth zones around small primary forest fragments, and rewilding efforts.

## Abstract (es)

Análisis de las perturbaciones en los bosques primarios sobrevivientes de Europa

**Resumen:**

Los bosques primarios son de suma importancia para la conservación de la biodiversidad y la prestación de servicios ambientales. En Europa, estos bosques son escasos y están amenazados por las actividades humanas. Sin embargo, no existe un análisis exhaustivo de la magnitud de las perturbaciones en estos bosques, en parte por su cartografía incompleta. Nuestro objetivo es proporcionar un análisis sistemático de las alteraciones en los bosques primarios de Europa con base en imágenes de teledetección desde 1986 hasta 2020. Evaluamos el área total perturbada, la tasa de área perturbada y la severidad de la perturbación, a nivel de país, continental y biogeográfico. Se utilizaron mapas de bosques primarios potenciales para llenar los vacíos en los mapas de los bosques primarios documentados. Observamos un aumento generalizado y significativo de las tasas de perturbación de los bosques primarios en toda Europa y un aumento de la gravedad de las perturbaciones en muchas regiones biogeográficas. Estos resultados son coherentes con la evidencia actual y asocian el declive actual de los bosques primarios en Europa con la actividad humana en muchas jurisdicciones. Si consideramos la limitada extensión de los bosques primarios en Europa y el alto riesgo de que se sigan perdiendo, es necesario tomar medidas urgentes y decisivas para garantizar la protección estricta de los restos de estos bosques de valor incalculable. Esto incluye el establecimiento de áreas protegidas alrededor de los bosques primarios, la expansión de zonas de crecimiento antiguo alrededor de pequeños fragmentos de bosque primario y esfuerzos de recuperación de la vida silvestre.

## INTRODUCTION

Primary forests are important for biodiversity and the provision of ecosystem services (Mikoláš et al., 2023; Paillet et al., 2010; Potapov et al., 2017; Sabatini et al., 2021a). These forests are home to endangered and endemic species (Eckelt et al., 2018), offer refugia (Vandekerkhove et al., 2011), and provide critical climate regulation services (Dellasala et al., 2020; Keith et al., 2009). For instance, they hold large amounts of carbon in their living biomass, deadwood, and carbon‐rich soils (Barredo et al., 2023; Hua et al., 2022; Keith et al., 2024; Potapov et al., 2017). Additionally, they supply fresh water, regulate hydrological cycles and local climate regimes, and contribute to the maintenance of human health (Watson et al., 2018). These forests have climate buffering capacity, producing an insulating effect during times of high temperatures (Frey et al., 2016). This contributes to their role as buffers against species loss (Di Marco et al., 2019).

Europe has the largest human footprint on Earth (Venter et al., 2016); thus, intact ecosystems are scarce. After millennia of forest use and exploitation (Kaplan et al., 2009), there are little primary forests in Europe (Barredo et al., 2021; Sabatini et al., 2018, 2021a). Moreover, Europe has the least amount of primary forests globally (FAO, 2020a). European primary forests are rare, small, and fragmented (Bas, 2020) and are subject to multiple disturbances and pressures (Mikoláš et al., 2023, 2019; Sabatini et al., 2021a; Thorn et al., 2018, 2017). The scarcity and uniqueness of primary forests in Europe further increase their conservation value (Mittermeier et al., 2003). This is recognized in the EU Biodiversity Strategy to 2030 (European Commission, 2020) and the New EU Forest Strategy for 2030 (European Commission, 2021), which call to define, map, monitor, and strictly protect all primary and old‐growth forests in the European Union (EU).

Despite its acknowledged importance, major data gaps exist regarding primary forests in Europe (Barredo et al., 2021; Sabatini et al., 2018, 2021a). It is estimated that in the EU, about 4.4 million ha of primary forests have not been systematically mapped, which is an area bigger than the Netherlands. The mapped area is estimated to cover about 1.35 to 3.2 million ha (Barredo et al., 2021). Significant information on disturbances in primary forests is also limited, which is a critical issue considering that these forests are declining across Europe (Mikoláš et al., 2023; O'Brien et al., 2021). Despite some efforts (e.g., Sabatini et al., 2021a), a comprehensive assessment of the magnitude of disturbances in primary forests in Europe is lacking, due in part to their incomplete mapping.

Natural disturbances are important drivers of ecosystem dynamics and help maintain biodiversity in primary forests. In contrast, anthropogenic actions, such as harvesting, salvage logging (postdisturbance logging), and prevention of natural disturbances, are drivers of habitat loss for many forest specialist species and forest communities (Mikoláš et al., 2017, 2019; Müller et al., 2019; Thorn et al., 2017). The definition of *primary forest* specifies that these forests show no indication of human activities and that their ecological processes are not significantly disturbed (FAO, 2018). This means that primary forests evolve within a natural disturbance regime, free from direct human influence. This definition has consequences for delineating and mapping primary forests. In particular, forests with indications of human disturbance are not included in maps of primary forests because they do not comply with this definition. This means that only those forests exhibiting no signs of human disturbances are included on these maps. One practical consequence of this situation is that assessing disturbances based on maps of primary forests is challenging due to the implicit selection bias. This occurs because primary forests that were anthropogenically disturbed before the mapping are omitted in the maps and therefore in disturbance accountings. As a result, the accounting would always represent a lower bound because those primary forests were anthropogenically disturbed before the mapping and were thus excluded.

In an effort to fill data gaps in primary forests and to provide the most comprehensive assessment possible, in addition to data on documented primary forests, we incorporated data on potential primary forests in our analysis. Although caution is necessary when interpreting data on potential primary forests, they can improve understanding of disturbances in areas with significant gaps in documented primary forest data, such as in Sweden. However, we analyzed the data sets on potential primary forests separately from those on documented primary forests.

We aimed to bridge the knowledge gap regarding disturbances in Europe's primary forests. To do so, we assessed disturbances in primary forests by adopting an approach that differentiates between the background rate of natural disturbances and the disturbance rate following the year of primary forest mapping, thereby yielding insights into the attribution of these disturbances to either natural causes or anthropogenic factors. We also evaluated disturbances in potential primary forests and examined data gaps in primary forests by comparing the combined extent of primary and potential primary forests with data from international reporting. Given the decline of Europe's remaining primary forests due to human activities, our assessment is timely and relevant.

We used state‐of‐the‐art remote sensing data on forest disturbances and up‐to‐date maps of primary and potential primary forests in Europe. The integration of this information delivered information useful for evaluating the condition of primary forests and identifying disturbance hotspots in primary forests across countries and biogeographical regions of Europe. This information can inform the conservation and protection of primary forests.

## METHODS

We used spatially explicit data on forest disturbances and primary forests across Europe (excluding the Russian Federation). We focused on EU countries and an additional 13 European countries (Albania, Andorra, Belarus, Bosnia and Herzegovina, Liechtenstein, Moldova, Montenegro, North Macedonia, Norway, Serbia, Switzerland, Ukraine, and the United Kingdom), resulting in a total of 40 countries.

### Forest disturbances maps

We used data from the European forest disturbance map (Senf, 2021; Senf & Seidl, 2021). This data set consists of maps of annual forest disturbances derived from Landsat satellite imagery covering Europe. The disturbance map covers the period 1986–2020 and indicates the year of the disturbance event on each grid cell. That is, grid cells are coded as either disturbed or undisturbed for each year. This map is complemented by a map of disturbance severity from 1986 to 2016 (Senf & Seidl, 2020). Severity measures the effect of disturbance in a continuous range from 0 to 1, where 0 indicates no change in forest canopy and 1 complete loss of canopy. Intermediate values represent different degrees of canopy loss. In addition, a forest mask is provided describing forest and nonforested land. All these data sets are disseminated at a 30 m grid size. The disturbance map has an overall accuracy of 87.6% (SE 0.5), and the mean absolute error of the assigned disturbance year was 3 years. The map of disturbance severity can faithfully delineate at least 3 classes of severity: undisturbed areas, nonstand‐replacing disturbances, and stand‐replacing disturbances.

### Map of primary forest

Data on primary forests were sourced from the European Primary Forest Database (EPFD) 2.0 (Sabatini et al., 2021a, 2021b). This is a geospatial database of primary forests that harmonizes 48 different, mostly field‐survey‐derived data sets (Appendix S1, panel A). The database describes 18,411 individual patches represented by unique nonoverlapping polygons and 299 point features covering an area of 3.7 million ha in Europe, excluding the Russian Federation. Data are from peer‐reviewed scientific literature or based on field checks conducted by trained researchers and professionals, suggesting high data reliability. The minimum mapping unit was 0.5 ha. Of the 299 point features, 78 had no information on forest patch area, and the remaining points together represented an extent lower than 70,000 ha, or 1.9% of the extent of primary forests in the complete database (excluding the Russian Federation). Therefore, considering that the delineation of primary forests corresponding to the point features was not available and that a large proportion of point features provided no information on extent, we focused only on data represented by polygon features. A map describing the primary forests in EPFD 2.0 is in figure 1 of Sabatini et al. (2021a).

Some data sets on primary forests covering parts of Hungary, Austria, and Switzerland had restricted access; therefore, they were not part of the digital file of EPFD 2.0. The UN's Educational, Scientific and Cultural Organization data set Ancient and Primeval Beech Forests of the Carpathians and Other Regions of Europe is listed in EPFD 2.0 and was sourced directly from the custodian due to copyright rules (Kirchmeir & Kovarovics, 2016; UNEP‐WCMC, 2021).

The Food and Agriculture Organization (FAO, 2018, p. 8) definition of primary forest is adopted in EPFD 2.0 and hence in our study. These forests have been defined as “naturally regenerated forest of native tree species, where there are no clearly visible indications of human activities and the ecological processes are not significantly disturbed.” The EPFD 2.0 includes patches of primary forests with different levels of naturalness, including old‐growth forests, late‐successional forests, and naturally regenerating forests after disturbances without subsequent management (Sabatini et al., 2021a).

The EPFD 2.0 data were compared with country‐level statistics on primary forests and assessed in relation to the disturbed area by Sabatini et al. (2021a). Their results suggest, first, an underestimation of primary forest area in EPFD 2.0 in relation to country statistics aggregated at the European level. However, in specific cases, there may be more primary forest area mapped in EPFD 2.0 than that reported by a given country. Second, they showed that EPFD 2.0 contains 1077 anthropogenically disturbed polygons, which corresponded to 6.2% of the total number of polygons (including the Russian Federation). These polygons were concentrated in the Russian Federation, southern Finland, and the Carpathians.

### Map of potential primary forest

We created a map of potential primary forests with data sets collected from 5 sources (Table 1). The data sets were identified in a previous data survey conducted in Europe (Barredo et al., 2021). Data sets 1 and 2 are part of EPFD 2.0; therefore, they are considered validated (confirmed) data sets. New areas of primary forests have been mapped in these 2 data sets since their inclusion in EPFD 2.0. Therefore, we included the new polygons in the map of potential primary forests.

**TABLE 1:** Data sets used for creating the map of potential primary forests in Europe.

Data set number	Source, country	Description	Number of polygons	Total area (ha)
1	WWF (World Wildlife Fund), old‐growth forests in Bulgaria	Describes verified old‐growth forest in Bulgaria and certain zones of Greece, Macedonia, and Serbia; data from extensive surveys and GIS mapping started in 2016 (https://www.wwf.mg/en/governance/?uNewsID=363462; https://gis.wwf.bg/mobilz/en/; https://naukazagorata.files.wordpress.com/2014/09/ng‐1‐2_2013_2.pdf)	18,170	113,821
2	WWF, virgin and quasi‐virgin forests in Romania	Verified data set built in collaboration with Romanian national authorities with data sets from orthophotos and field surveys; WWF used previous information collected by the Pin Matra project (Biriş & Veen, 2005), which was updated and verified (https://www.wwf.mg/oceans_footer/?uNewsID=335430; https://lemncontrolat.ro)	4096	62,908
3	Greenpeace, potential primary forests map of Romania	Estimation of location and extent of potential primary forests in Romania; created using remote sensing and GIS techniques (Ibisch & Ursu, 2017) (https://maps.greenpeace.org/project/potential‐primary‐forests‐map‐of‐romania)	5986	295,602
4	PRIMOFARO, inventory of potential primary and old‐growth forests in Romania	Data from visual analyses of satellite and aerial images; existing inventories of primary forest assessed for mapping intact remains; final data set identified over 0.5 million ha of potential primary and old‐growth forests in Romania (Schickhofer & Schwarz, 2019); data set includes areas from Romania and Ukraine	7624	509,906
5	Sabatini et al. (2021a), map of potential primary forests in Sweden	EPFD 2.0 includes data sets of potential primary forests for Sweden and Norway (Sabatini et al., 2021a); data for Sweden covers 2.4 million ha; polygons are an approximation of the primary forest area without ground validation (Sabatini et al., 2021a, 2021b)	14,300	2,367,655
6	Sabatini et al. (2021a), map of potential primary forests in Norway	Similar to data set 5; polygons of potential primary forest for Norway are an approximation without ground validation (Sabatini et al., 2021a, 2021b)	2104	139,030

For integrating the 6 data sets, we followed the definition framework of Sabatini et al. (2021a) and Sabatini et al. (2018). The 6 data sets were merged in one map eliminating overlaps and unifying adjacent polygons. Then, areas overlapping polygons of primary forests in EPFD 2.0 were eliminated from the map of potential primary forests. The resulting map contained 37,969 nonoverlapping polygons representing a total area of 3.2 million ha (Appendix S1, panel B).

### Forest disturbance indicators

Indicators of disturbed area and disturbance severity in primary forests and potential primary forests were calculated based on the annual disturbance maps (Senf, 2021; Senf & Seidl, 2021), following Johnstone et al. (2016), Senf and Seidl (2021), and Turner (2010). We used a minimum mapping unit of 2 grid cells, or 0.18 ha, following Senf and Seidl (2021). We used a minimum mapping unit to remove unrealistic outliers from the maps. Therefore, we removed all disturbance patches smaller than 0.18 ha from the disturbance maps, as well as all forest cover patches smaller than this size from the forest mask. Patches were delineated using rook contiguity, as in Senf and Seidl (2021).

We calculated the area disturbed in primary forests before and after the year when each primary forest data set in EPFD 2.0 (Sabatini et al., 2021a) was surveyed, that is, the mapping year for each data set. Information on the mapping year of the data sets was sourced from the data set overview document accompanying the database (Sabatini et al., 2021b). We assumed that all disturbances occurring before or during the mapping year (hereafter predating) were attributable to natural causes. Otherwise, the primary forest polygon would not have been delineated as such because it would no longer meet the criteria for being considered a primary forest. Disturbances occurring after the mapping year (hereafter postdating), in contrast, could be either natural or anthropogenic. Thus, the disturbed area predating and postdating the mapping year was determined by calculating the area of the disturbance grid cells in polygons of primary forests before and after the mapping year of each data set during 1986–2020 (i.e., the period of the disturbance data).

The indicator was expressed in 3 ways: total area disturbed, proportion of area disturbed, and mean annual rate of area disturbed. These were represented in the periods predating and postdating the mapping year. The area of primary forest was calculated based on the forest area according to the forest mask that fell within polygons of primary forests.

We performed a sensitivity analysis to determine the impact of potential variations in the mapping year of the primary forest data sets on the mean annual rate of disturbance in each country, the EU, and at the European level.

Disturbance severity was calculated using the disturbance severity map (Senf, 2021; Senf & Seidl, 2021). Mean severity was calculated at patch level for each year, country, and biogeographical region with disturbance patches that fell in polygons of primary forests. The mean severity was calculated at the patch level as the mean severity of all disturbance grid cells on a disturbed patch. Then, the mean of all patches was aggregated at country and biogeographical region levels.

All the indicators were aggregated at the country and biogeographical region (EEA, 2016). The Alpine biogeographical region was split into 2 subregions because of the major environmental differences between the Alpine Scandinavian and the rest of the Alpine zones in more southerly latitudes, following Maes et al. (2023). Therefore, one subregion represented the Alpine Scandinavian and the other the rest of the Alpine subregion, that is, the mountain ranges of the Alps, Apennines, Pyrenees, and Carpathians. The indicators were also aggregated at the EU and European level following the approaches described above but aggregating the results to these 2 macroregions.

A similar approach was used to calculate the indicators for potential primary forests. However, in this instance, attributing disturbances to the year before or after the mapping year was not feasible due to the potential nature of the data sets. That is, these data sets identify areas potentially hosting primary forests, which implies that there is no reference year available to define the end of the natural disturbance regime. For this reason, for potential primary forests, the indicators were calculated for the entire disturbance period from 1986 to 2020.

Equal‐area projection maps were used for area calculations, taking the curvature of the Earth into consideration. Available data on forest disturbances covered all the polygons of primary and potential primary forests, except the territories of the Canary Islands (Spain), Madeira Islands, and Azores (Portugal). Therefore, we did not include these territories.

We calculated 95% confidence intervals (CIs) for the area disturbed, mean annual rate of area disturbed, and mean severity, with a bootstrapping with replacement approach over 10,000 iterations. We employed a nonparametric Mann–Whitney *U* test on samples with a maximum size of 1000 patches, selected to avoid spatial autocorrelation, to determine whether there was a statistically significant difference between the means of mean patch disturbance severity before and after the mapping year in primary forests. We used a nonparametric bootstrapping with replacement approach over 10,000 iterations to assess whether there was a statistically significant difference between the mean annual rates of area disturbed in primary forests predating and postdating the mapping year. Details are in Appendix S2.

### Comparison of country‐level information

Validation of the delineated areas for primary and potential primary forests would require extensive ground‐level information, which is not readily available in Europe. Previously, Sabatini et al. (2021a) validated EPFD 2.0 by comparing the extent of primary forests against country‐level data from the Forest Europe initiative (FOREST EUROPE, 2020) and the Global Forest Resource Assessment (FRA) (FAO, 2020a). We adopted the same approach to assess the completeness of the collected maps. However, a key difference from the validation conducted by Sabatini et al. (2021a) is that here we assessed the difference between the combined extent of primary and potential primary forests and the area of primary forests reported in the FRA at the country level. We accounted for the combined area of primary and potential primary forests solely for the purpose of the comparison with FAO data. Apart from this, the 2 types of data were assessed separately in all the other sections of this study. Additionally, a second assessment was conducted in countries hosting potential primary forests with, in this case, ancillary information from peer‐reviewed literature and documented reports, in addition to the FRA.

## RESULTS

### Disturbances in primary forests

Of the total 2.4 million ha of primary forests in Europe, 61,836 ha (95% CI 53,402–70,270) or 2.6% were disturbed before the mapping year of the primary forest data sets. After the mapping year, the area disturbed was 14,388 ha (95% CI 11,781–16,995) or 0.6%. In the EU area, the figures exhibited a similar pattern. Of the total 2 million ha of primary forests, 42,347 ha (95% CI 35,932–48,762) or 2.1% were disturbed before the mapping year and 10,585 ha (95% CI 7965–13,205) or 0.5% were disturbed after the mapping year (Table 2). The imbalance between the amount of primary forest area disturbed before and after the mapping year was expected because the mean mapping year of the polygon data sets (weighted by the area of the polygons) in EPFD 2.0 is 2015. Therefore, this year is closer to the last year of the disturbance data series, 2020, than to the initial year, 1986, which creates the imbalance. The mean annual rate of area disturbed provided a metric that adjusted for the imbalance. The disturbance distribution yielded mean rates of primary forest disturbance of 0.08% (95% CI 0.03–0.14) per year and 0.15% (95% CI 0.08–0.23) per year in Europe for the periods predating and postdating the mapping year, respectively. This yielded a significant increase of 0.07% (*p* < 0.05) per year between the 2 periods (Table 2), which suggests that the percentage of primary forest area disturbed increased after the mapping year of the data sets. In the EU, the mean rate increased by 0.04% (*p* < 0.10) per year, from 0.07% (95% CI 0.02–0.12) to 0.10% (95% CI 0.05–0.16) for the same respective periods (note the rounding effect).

**TABLE 2:** For primary forest (PF) in Europe, area, proportion of forest area, area disturbed, and mean annual rate of area disturbed predating (including mapping year) and postdating the primary forest's mapping year by biogeographical region from 1986 to 2020.

Biogeographical region	Area (thousands of ha)	Proportion of total forest area^a^ (%)	Area disturbed (thousands of ha) predating the mapping year (95% CI)	Mean rate of PF area disturbed (%/year) predating the mapping year (95% CI)	Area disturbed (thousands of ha) postdating the mapping year (95% CI)	Mean rate of PF area disturbed (%/year) postdating the mapping year (95% CI)	Change in mean rate of PF area disturbed (%/year)^b^
Alpine	216	0.9	4.9 (4.3–5.5)	0.08 (0.04–0.11)	2.9 (2.4–3.4)	0.32 (0.26–0.39)	0.24**
Alpine–Scandinavian	506	5.4	4.0 (2.6–5.2)	0.03 (0.02–0.04)	0.8 (0.6–1.0)	0.04 (0.02–0.07)	0.02
Anatolian	No data	–	–	–	–	–	–
Arctic	0	–	–	–	–	–	–
Atlantic	14	0.1	0.3 (0.2–0.4)	0.07 (0.04–0.10)	0.1 (0.0–0.1)	0.14 (0.11–0.18)	0.08
Black Sea	7	1.5	<0.1 (0.0–0.1)	0.02 (0.00–0.04)	<0.1 (0.0–0.1)	0.08 (0.00–0.18)	0.06
Boreal	1480	2.1	38.0 (31.4–43.1)	0.08 (0.03–0.14)	7.5 (5.1–10.5)	0.12 (0.08–0.16)	0.04
Continental	167	0.2	14.4 (8.6–19.6)	0.27 (0.14–0.40)	3.1 (2.1–4.3)	0.58 (0.32–0.84)	0.31
Macaronesia	No data	–	–	–	–	–	–
Mediterranean	10	<0.1	0.3 (0.2–0.3)	0.09 (0.02–0.15)	0.1 (0.0–0.2)	0.24 (0.00–0.81)	0.15
Pannonian	1	<0.1	<0.1 (0.0 to <0.1)	0.08 (0.00–0.24)	<0.1 (0.0 to <0.1)	0.39 (0.33–0.45)	0.32**
Steppic	<1	<0.1	0	–	0	–	–
Total in Europe	2401	1.1	61.8 (53.4–70.3)	0.08 (0.03–0.14)	14.4 (11.8–17.0)	0.15 (0.08–0.23)	0.07**
Total in EU	1999	1.1	42.3 (35.9–48.8)	0.07 (0.02–0.12)	10.6 (8.0–13.2)	0.10 (0.05–0.16)	0.04*

^a^ According to the forest mask of Senf and Seidl (2021).

^b^ Mean rate of PF area disturbed postdating the mapping year minus mean rate of PF area disturbed predating the mapping year.

Significance: *p < 0.10; **p < 0.05.

Among the biogeographical regions, 2 exhibited a significant (*p* < 0.05) increase in the mean rate of area disturbed between the predating and postdating periods. These increases were 0.32% and 0.39% per year in the Alpine and Pannonian regions, respectively. All other regions exhibited increases in the mean rate of primary forest area disturbed, although these were not statistically significant. The Boreal region had the most primary forest in Europe and the largest disturbed area in the postdating period, accounting for 7.5 thousand ha (95% CI 5.1–10.5). This finding is consistent with the fact that the Boreal region contains the largest extent (62%) of European primary forests among the biogeographical regions.

Mean disturbance severity, which refers to the probability of complete canopy loss at the patch level, was generally high in primary forests. Mean severity was 0.67 and 0.68 in the predating and postdating periods, respectively, in Europe. This indicates severity was high and stable during both periods. In the EU region, severity also exhibited a similarly stable pattern with no significant change between periods (Table 3). However, the magnitude of disturbance severity across biogeographical regions differed between predating and postdating periods. In particular, the Pannonian, Black Sea, Alpine–Scandinavian, and Mediterranean regions exhibited significant increasing changes in mean severity (*p* < 0.05). The increase was especially pronounced in the Alpine–Scandinavian region, where severity rose from 0.66 to 0.77, marking the highest severity across the regions in the postdating period, which may suggest the occurrence of stand‐replacing disturbances in this period.

**TABLE 3:** For primary forest in Europe, mean patch disturbance severity (range 0–1) predating (including mapping year) and postdating the primary forest's mapping year in European biogeographical regions from 1986 to 2016.

Biogeographical region	Mean severity predating the mapping year (95% CI)	Mean severity postdating the mapping year (95% CI)	Change in mean severity between the predating and postdating mapping year
Alpine	0.63 (0.63–0.64)	0.64 (0.63–0.64)	0.01
Alpine–Scandinavian	0.66 (0.66–0.67)	0.77 (0.76–0.78)	0.10^*^
Anatolian	No data (ND)	ND	ND
Arctic	–	–	–
Atlantic	0.75 (0.74–0.77)	0.69 (0.66–0.72)	−0.06^*^
Black Sea	0.43 (0.39–0.48)	0.57 (0.45–0.67)	0.13^*^
Boreal	0.69 (0.69–0.69)	0.68 (0.68–0.69)	−0.01^*^
Continental	0.66 (0.66–0.66)	0.65 (0.64–0.66)	−0.01
Macaronesia	ND	ND	ND
Mediterranean	0.60 (0.58–0.62)	0.64 (0.58–0.71)	0.04^*^
Pannonian	0.50 (0.42–0.57)	0.63 (0.54–0.74)	0.14^*^
Steppic	–	–	–
Total in Europe	0.67 (0.67–0.68)	0.68 (0.68–0.68)	0.0046
Total in EU	0.68 (0.68–0.68)	0.68 (0.68–0.68)	−0.0002

^*^ *p* < 0.05.

**FIGURE 1:**
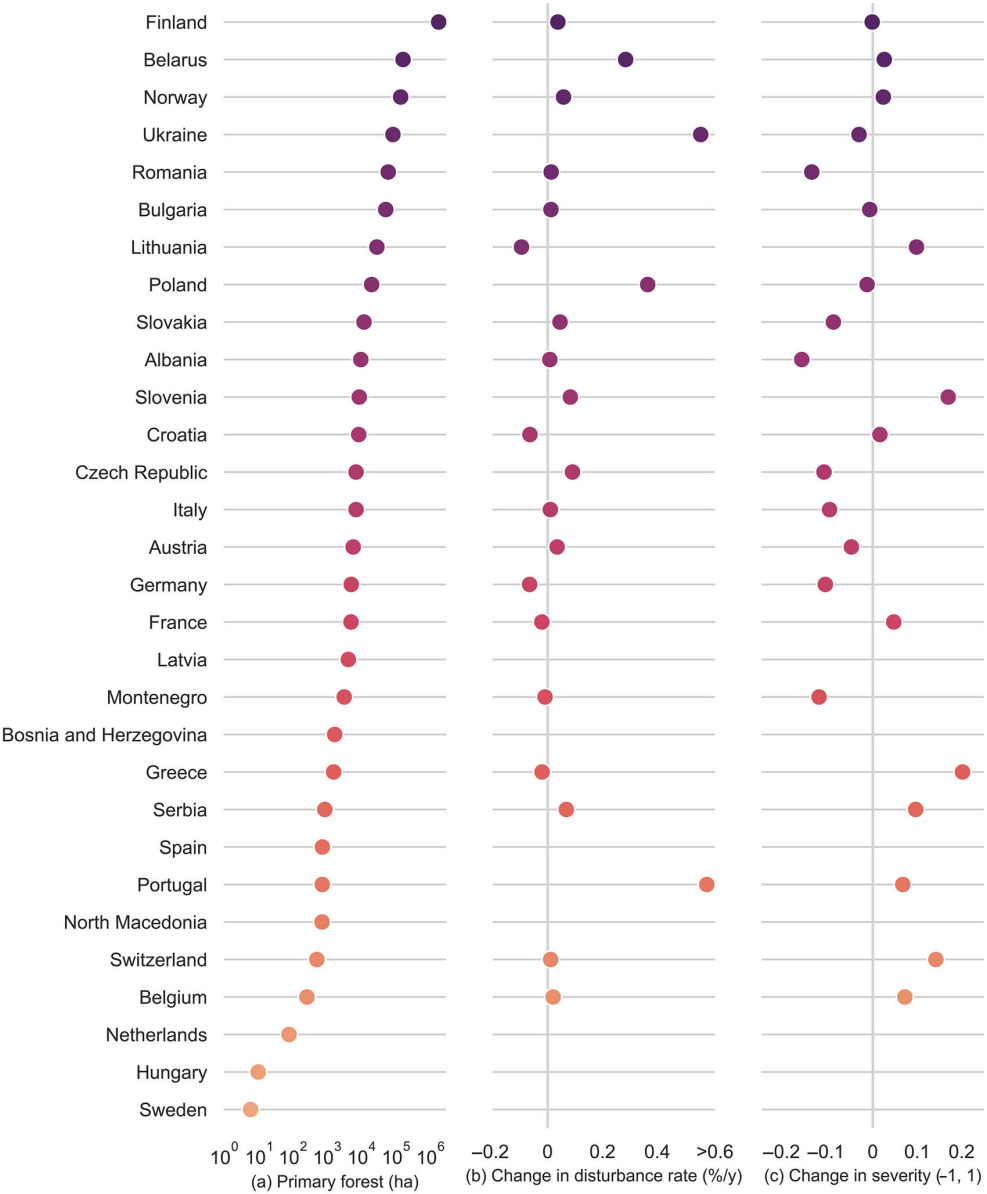
For primary forest in Europe: (a) area, (b) change in mean annual rate of area disturbed, and (c) change in mean patch disturbance severity from the period predating (including mapping year) to the period postdating the primary forest's mapping year in European countries from 1986 to 2020, except for severity, which is 1986–2016 (vertical gray lines, zero or no change; left‐most graph is on a log scale).

Seven countries exhibited a significant increase in the mean rate of primary forest area disturbed between the predating and postdating periods (*p* < 0.05 or 0.10) (Figure 1; Appendix S3). The most significant changes were observed in Ukraine, Poland, and Portugal, although the area of primary forest in Portugal was low (around 730 ha). In these countries, the mean rate of primary forest area disturbed in the postdating period was substantially greater than the mean for the European region of 0.15% (95% CI 0.08–0.23) per year. The other 4 countries, Finland, Norway, Serbia, and Slovenia, all exhibited changes greater than 0.04% per year. These 7 countries together contain 84% and 90% of the primary forests in Europe and the EU, respectively.

The observed significant shifts in the mean rate of primary forest area disturbed between the predating and postdating periods may suggest the occurrence of major disturbances in more recent times (postdating period), which have contributed to the significant increases in the rates. The case of Sweden is unusual because data on documented primary forests were nearly absent from the polygon data set we used. Results for this country are discussed later in the section on potential primary forests. Results of the sensitivity analysis are in Appendices S4–S6.

Changes in disturbance severity varied among countries (Figure 1; Appendix S7). Five countries had significant (*p* < 0.05) increases in severity between the predating and postdating periods: Belarus, Lithuania, Norway, Portugal, and Slovenia. Eleven countries exhibited an opposite significant change. This indicated wide variability in disturbance severity among countries. Some other cases that showed minor or no significant changes in severity still exhibited high severity in both periods: Belgium, Finland, France, Poland, and Ukraine.

### Disturbances in potential primary forests

**FIGURE 2:**
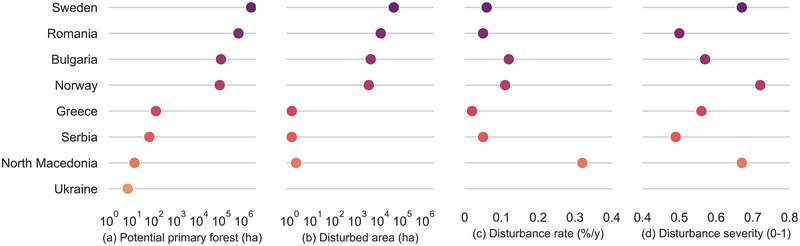
For potential primary forest in Europe: (a) area, (b) disturbed area, (c) mean annual rate of area disturbed, and (d) mean patch disturbance severity in European countries from 1986 to 2020, except for severity, which is 1986–2016 (first 2 graphs are on the log scale).

The extent of potential primary forests was estimated at 2.94 and 2.86 million ha in Europe and the EU, respectively. These figures represent 1.3% and 1.6% of the total forest area in both regions. The extent of potential primary forests in Europe exceeded that of documented primary forests by approximately 23%, or 543,000 ha. Just as with primary forests, the Alpine–Scandinavian, Boreal, and Alpine biogeographical regions had most of the potential primary forests in Europe, accounting together for 91% of their total area (Table 4). Only Sweden, Romania, Bulgaria, and Norway accounted for nearly all of the potential primary forests in Europe (Figure 2; Appendix S8).

**TABLE 4:** For potential primary forest (PPF) in Europe, total area, proportion of total forest area, area disturbed, mean annual rate of area disturbed, and mean patch disturbance severity.

Biogeographical region	Area (thousands of ha)	Proportion of total forest area (%)^a^	Area (thousands of ha) disturbed 1986–2020 (95% CI)	Proportion of PPF area disturbed 1986–2020 (%)	Mean rate (%/year) of PPF area disturbed 1986–2020 (95% CI)	Mean severity (range 0–1) (95% CI)^b^
Alpine	463	1.9	10.9 (7.2–16.6)	2.4	0.07 (0.04–0.10)	0.52 (0.51–0.52)
Alpine–Scandinavian	1320	14.2	19.2 (10.5–30.7)	1.5	0.04 (0.02–0.07)	0.65 (0.64–0.65)
Anatolian	No data (ND)	–	–	–	–	–
Arctic	0	–	–	–	–	–
Atlantic	26	0.1	0.5 (0.4–0.7)	2.1	0.06 (0.05–0.08)	0.68 (0.67–0.69)
Black Sea	11	2.5	0.4 (0.2–0.5)	3.2	0.09 (0.06–0.14)	0.43 (0.41–0.45)
Boreal	905	1.3	26.0 (20.1–32.7)	2.9	0.08 (0.06–0.10)	0.70 (0.69–0.70)
Continental	214	0.3	3.9 (3.0–4.9)	1.8	0.05 (0.04–0.07)	0.51 (0.50–0.53)
Macaronesia	ND	–	–	–	–	–
Mediterranean	<1	<0.1	<0.1 (0.00–0.01)	6.2	0.18 (0.06–0.37)	0.65 (0.41–0.84)
Pannonian	2	<0.1	<0.1 (0.00–0.02)	0.7	0.02 (0.00–0.04)	0.49 (0.38–0.60)
Steppic	3	0.1	0.1 (0.08–0.14)	3.2	0.09 (0.07–0.12)	0.59 (0.55–0.62)
Total in Europe	2943	1.3	61.0 (44.9–80.4)	2.1	0.06 (0.04–0.08)	0.64 (0.63–0.64)
Total in EU	2857	1.6	57.7 (41.9–76.7)	2.0	0.06 (0.04–0.08)	0.63 (0.63–0.63)

^a^ According to the forest mask of Senf and Seidl (2021).

^b^ Period 1986–2016.

Our inability to associate a specific mapping year with the data sets of potential primary forests led to an assessment that encompassed the entire series of disturbances from 1986 to 2020. The disturbed area of potential primary forests accounted for in Europe was 61,003 ha (95% CI 44,905–80,408), or 2.1%, of which the disturbed area in the EU was 57,675 ha (95% CI 41,915–76,677), or 2%.

The Boreal region exhibited the highest amount of disturbed area, 26,000 ha (95% CI 20,100–32,700), or 2.9%. It was followed by the Alpine–Scandinavian and the Alpine regions, with 19.2 thousand ha (95% CI 10,500–30,700), or 1.5%, and 10.9 thousand ha (95% CI 7200–16,600), or 2.4%, respectively. Of these 3 regions, the Boreal region exhibited the highest mean rate of disturbance at 0.08% (95% CI 0.06–0.10) per year, which is higher than the rate in Europe at 0.06% (95% CI 0.04–0.08) per year (Table 4). Likewise, the mean severity of 0.70 (95% CI 0.69–0.70) in the Boreal region was the highest in Europe and may suggest a predominance of stand‐replacing disturbances. Similarly, the Atlantic and Alpine–Scandinavian regions showed high mean severities of 0.68 (95% CI 0.67–0.69) and 0.65 (95% CI 0.64–0.65), respectively. Across all regions, the Mediterranean showed the highest mean rate of potential primary forest disturbed, 0.18% (95% CI 0.06–0.37) per year, along with a high disturbance severity of 0.65 (95% CI 0.41–0.84). However, this region had <1000 ha of these forests, which is <0.1% of its forested area.

Most of the disturbed area of potential primary forests in Europe was concentrated in Sweden and Romania, which accounted for 88% of the total disturbed area, equivalent to 53,647 ha (Figure 2; Appendix S8). However, among the 4 countries hosting large extents of potential primary forests (more than 50,000 ha), Bulgaria and Norway exhibited the highest mean rates of disturbance, 0.12% (95% CI 0.10–0.14) and 0.11% (95% CI 0.09–0.13) per year, respectively, well above the mean rate for Europe, which is 0.06% (95% CI 0.04–0.08) per year. In addition, Norway also exhibited high disturbance severity at 0.72 (95% CI 0.71–0.72), followed by Sweden at 0.67 (95% CI 0.67–0.67). The disturbance severity in these 2 countries was substantially higher than in other countries, such as Bulgaria or Romania, which had severities of 0.57 (95% CI 0.57–0.58) and 0.50 (95% CI 0.49–0.50), respectively.

### Comparison of combined mapped area of primary and potential primary forests and ancillary sources

There were significant discrepancies with respect to FRA on a country‐by‐country basis. Data for 3 countries that reported having primary forests in excess of 75,000 ha were not included on the maps we used. Our area calculations were lower than those reported in the FRA for 11 countries, resulting in a collective shortfall of over 964,000 ha (see Appendix S9 for the detailed assessment).

For the 4 countries with the majority of potential primary forests for which we compared the area we found with data from additional sources, there were notable discrepancies. Our data showed overestimations for Romania, parity for Sweden and Norway, and an underestimation for Bulgaria (Appendices S9 & S10). Caution is advised when comparing different estimates of primary forests because calculation methods, data sources, and definitions varied and internal consistency is lacking in FRA data on primary forests (Mackey et al., 2021; O'Brien et al., 2021).

## DISCUSSION

We conducted a systematic assessment of disturbed area, annual rate of area disturbed, and patch disturbance severity in primary and potential primary forests in Europe, improving the knowledge base on the condition of these forests. Our most notable finding was the significant increased annual rate of area disturbed in primary forests in the postdating period across Europe and the EU. Likewise, increases in the rate were found across all biogeographical regions, 2 of which were statistically significant. The trend of significant increase held true for 7 countries that together represented 84% and 90% of the primary forests in Europe and the EU, respectively. The observed rise in the mean rate of primary forest area disturbed across these jurisdictions indicated an acceleration in the disturbance regime in the postdating period. This is further reinforced by observations of heightened disturbance severity in many biogeographical regions, such as the Alpine–Scandinavian region, which exhibited a mean patch disturbance value of 0.77 in the postdating period in contrast with 0.66 in the predating period, suggesting disturbances that result in almost complete canopy loss in the affected patches. Although there is evidence of shifts in natural disturbance regimes in Europe (Patacca et al., 2023), there is also growing evidence pointing to the occurrence of anthropogenic disturbances in primary forests, particularly logging, which represents a major threat to these ecosystems. Our results are consistent with this evidence.

Although the annual rate of primary forest area disturbed during the predating period can be safely attributed to natural disturbances, the disturbance rate in the postdating period may arise from a combination of natural and anthropogenic disturbances. We speculate that at least a portion of the increase in the rate between the 2 periods could be associated with direct anthropogenic disturbances (Table 2 and central panel in Figure 1). Although major natural disturbances in the postdating period may have contributed to increased rates of area disturbed in the countries or subregions where they occurred, the widespread nature of the observed changes suggests the occurrence of more pervasive drivers. In summary, the magnitude and extent of the change observed seemed difficult to reconcile with natural disturbances alone.

The consistent increase in the annual rate of area disturbed across all biogeographical regions, the EU, and Europe (Table 2) should be considered a warning signal. In fact, their persistence in time would imply a trajectory toward the destruction of these forests. This highlights the need for further monitoring of disturbance rates in primary forests in Europe.

The assessment of potential primary forests was limited due to the inability to assess disturbances before and after the mapping year. Despite this limitation, our results showed that the Boreal region had the highest mean rate of area disturbed (0.08% per year) among potential primary forests from 1986 to 2020. This rate is twice as high as that of the Alpine–Scandinavian region. The Boreal region also displayed a high mean severity of disturbance, suggesting the occurrence of stand‐replacing disturbances. These are consistent with clearcutting but may also align with natural disturbances, such as intense fires or strong windstorms. Other regions with disturbance rates exceeding the European mean of 0.06% per year include the Alpine (which encompasses the Carpathians), Black Sea, Mediterranean, and Steppic. Notably, Bulgaria and Norway exhibited significantly high mean rates of area disturbed in potential primary forests, above 0.11% per year. Likewise, Norway and Sweden had high levels of disturbance severity.

The rate and severity of disturbed primary and potential primary forests varied widely across countries (Figures 1 & 2). This reflects different climatic and ecological settings, varying levels of environmental protection (Barredo et al., 2021), compliance with protection, and use intensity of forest resources. Although determining whether the driver of the disturbance events was natural or anthropogenic warrants further research, our results are consistent with evidence indicating the decline of primary forests across Europe. Therefore, it is reasonable to suggest that a proportion of the 14,388 ha of primary forests disturbed after the mapping year of the primary forest data sets and the 61,003 ha of potential primary forests disturbed from 1986 to 2020 in Europe can be attributed to logging operations.

Our results are consistent with evidence from nongovernmental organizations and peer‐reviewed literature that suggest the ongoing decline of primary forests in Europe due to human action (Mikoláš et al., 2023; O'Brien et al., 2021). Their destruction has occurred in the Romanian Carpathians (Agent Green & EuroNatur, 2023; Agent Green et al., 2022; EuroNatur & Agent Green, 2019; Hurtes & Cai, 2022; Knorn et al., 2013; Luick et al., 2021), Slovak Republic (Mikoláš et al., 2019), Bulgaria (EIA, 2022; WWF, 2012), the Ukrainian Carpathians (Earthsight, 2018; Hrynyk, 2021), old‐growth boreal forests in Sweden and Finland (Aalto et al., 2022, 2020; Ahlström et al., 2022; Andersson, 2021; Greenpeace Nordic/Finland, 2022), and elsewhere (IUCN, 2020; Sabatini et al., 2021a). Compliance with protection measures is also a fundamental aspect of these forests. For instance, the EU's highest court ruled against illegal logging in primary forests in the Natura 2000 site surrounding Białowieża National Park in Poland (Court of Justice of the European Union, 2018; Müller et al., 2019; Schiermeier, 2018; Żmihorski et al., 2018).

Existing EU legislation does not explicitly prohibit logging in primary forests. However, restrictions apply to wood from these forests if logging leads to deforestation or forest degradation, as defined by the EU Deforestation Regulation (European Union, 2023b), or if the wood is intended for bioenergy use, in accordance with the EU Renewable Energy Directive (European Union, 2023a). Nevertheless, logging in primary forests is not expressly forbidden in the EU provided that it occurs outside strictly protected areas or areas protected by the EU Habitats Directive (European Union, 1992).

Policy makers should be aware that the loss of these ecosystems is irreversible and urgent steps must be taken to protect them and prevent further decline (Mikoláš et al., 2023). The proportion of known primary forests under strict protection is estimated at around 87% in the EU (Barredo et al., 2021). However, the situation for potential primary forests is more problematic because, to the best of our knowledge, their protection status is not known at a continental level. In Romania, for instance, Munteanu et al. (2022) estimated that <10% of potential primary forests are strictly protected. This confirms the urgent need for a comprehensive inventory of documented primary forests to assess their protection level and to outline the necessary actions to enable their strict protection in the EU.

Primary forests cover only a marginal 2.4 million ha, or 1.1% (Table 2), of the total forest extent in Europe (Sabatini et al., 2018, 2020, 2021a). This implies that the area reported in the FRA is 75% larger than the area of documented primary forests. However, we accounted for 2.9 million ha of potential primary forests in Europe that were almost entirely in the EU. Thus, the combined area of primary and potential primary forests totals 5.3 million ha, or 2.3% of the total European forest extent. This results in the area reported in the FRA being 21% smaller than the combined area of primary and potential primary forests we calculated.

In summary, the observed discrepancies with FRA data, especially the significant shortfall in documented primary forests, suggest that a large proportion of potential primary forests might correspond to actual primary forests. Furthermore, new mapping efforts in some Balkan countries, for example, Bosnia and Herzegovina and Montenegro, could lead to a higher documented presence of primary forests in this region (Motta et al., 2024). Nevertheless, comparing the area of primary forests we calculated with the FRA data was challenging. First, the information on primary forests in the FRA is subject to uncertainties due to the considerable variation in how countries apply the definition and the different approaches used to calculate the area (FAO, 2020b; Mackey et al., 2021; O'Brien et al., 2021). Second, the differing definitions of *forest* used by the FRA and the forest mask we used to calculate area constrained comparability. Results of our assessment of potential primary forests underscore the need for more efforts to accurately map primary forests in Europe. A major aim should be to create ground‐validated, spatially explicit, comprehensive data on primary forests. This is a major requirement for more robust monitoring of the condition of these forests and is one of the aims of the EU Biodiversity Strategy for 2030 (European Commission, 2020).

Limitations of our study are associated with uncertainties in the data we used arising from, for example, data gaps, sampling errors, and data inaccuracies. Although EPFD 2.0 is the most comprehensive and up‐to‐date database on primary forests in Europe, its authors acknowledge the potential existence of bias and interpretation errors in the data sets used for its creation (Sabatini et al., 2021a). One concern pertains to the completeness of the mapped area of documented primary forests. Our use of maps of potential primary forests, some of which were sourced from EPFD 2.0, helped alleviate the mapping gaps regarding documented primary forests. However, it is important to exercise caution when interpreting the information derived from maps of potential primary forests because they lack ground‐level validation in certain regions.

Results of our comparison of the area of primary forests we calculated, FRA data, and data from additional sources suggest that, despite major differences in accounting approaches, the combined area of primary and potential primary forests we found seems reasonably consistent with the FRA and other sources in many countries. However, there are still gaps in the area accounted for in relation to the FRA, which may suggest underestimation in the maps of primary and potential primary forests in specific countries. In other cases, the situation was the opposite—for example, Finland reported 0.2 million ha of primary forests to the FRA, which contrasts with the 1.77 million ha accounted for in EPFD 2.0 (Appendix S3). This figure aligns with the 1.6 million ha reported by the Finnish Nature Panel (Kotiaho et al., 2021). Although further analyses and more comprehensive data are necessary for robust conclusions, our initial findings provide a strong basis for ongoing research.

When assessing our results, it is important to consider that many primary forests were logged before the mapping surveys (Mikoláš et al., 2023). This implies that the disturbed area in primary forests we accounted for refers only to surviving primary forests, that is, those forests where there are no clearly visible indications of human activities. In short, our assessment focused only on the remaining primary and potential primary forests, which represented only 2.3% of the total forest area in Europe. Additionally, polygons included in EPFD 2.0 were delineated (mapped) only after the year 2000, which is subsequent to the starting year of the disturbance maps, beginning in 1986. Consequently, we hypothesize that primary forests affected by logging between 1986 and the start of the mapping period for each data set were not included in EPFD 2.0 because they were no longer classified as primary following logging. This suggests the existence of a selection bias in the data on primary forests used, which implies that the actual area of disturbed primary forests might be larger than what we report.

Although information on the reliability of the mapping year is not provided in EPFD 2.0 (Sabatini et al., 2021a, 2021b), our sensitivity analysis suggests that potential uncertainties surrounding the mapping year of the primary forest data sets are unlikely to significantly alter our results (Appendix S4), at least within the 7 years assessed in the sensitivity analysis.

The risk of destruction of the remaining primary forests in Europe has been documented, which calls for an urgent need to strictly protect these highly valuable ecosystems. Primary forests that have not been protected and for which protection enforcement is inadequate can be lost. The European Commission has taken action pursuing the nonbinding aim of strictly protecting these forests by 2029, as called for in the Biodiversity Strategy to 2030 (European Commission, 2023). Apart from this, there is consensus among scientists that decisive and urgent action is needed for the strict protection of primary forests (Di Marco et al., 2019; Mikoláš et al., 2023; O'Brien et al., 2021). Necessary steps for their protection include the complete mapping of all primary forests, including the verification of data on potential primary forests; establishing monitoring systems using remotely sensed imagery combined with ground data; and defining and implementing strict protection regimes. This includes banning salvage logging (postdisturbance logging) (Müller et al., 2019) and delineating protected zones around primary forests.

## Supporting information



Supporting Information
