# Supplementary material for: Assessing disturbances in surviving primary forests of Europe
Source: Conserv Biol. 2024 Nov 19;39(2):e14404. doi: 10.1111/cobi.14404 (PMC11959323; doi:10.1111/cobi.14404)
Supplement: Supplementary file 1 — Supporting Information [file COBI-39-e14404-s001.docx]

**Appendix S1**. A) Example of primary forest, forest area, and disturbed forest data sets in western Romania. Data sources: Primary forest, EPFD 2.0 (Sabatini et al., 2021a; Sabatini et al., 2021b); Forest/non-forest and forest disturbances from 1986 to 2020, Senf and Seidl (2020, 2021). The small inset in the panel shows the location of the area. B) As for A, but for potential primary forest in Norway. Source of the potential primary forests data shown in B, EPFD 2.0 (Sabatini et al., 2021a; Sabatini et al., 2021b). Table 1 in the manuscript shows the other data sources of potential primary forest used in the study.


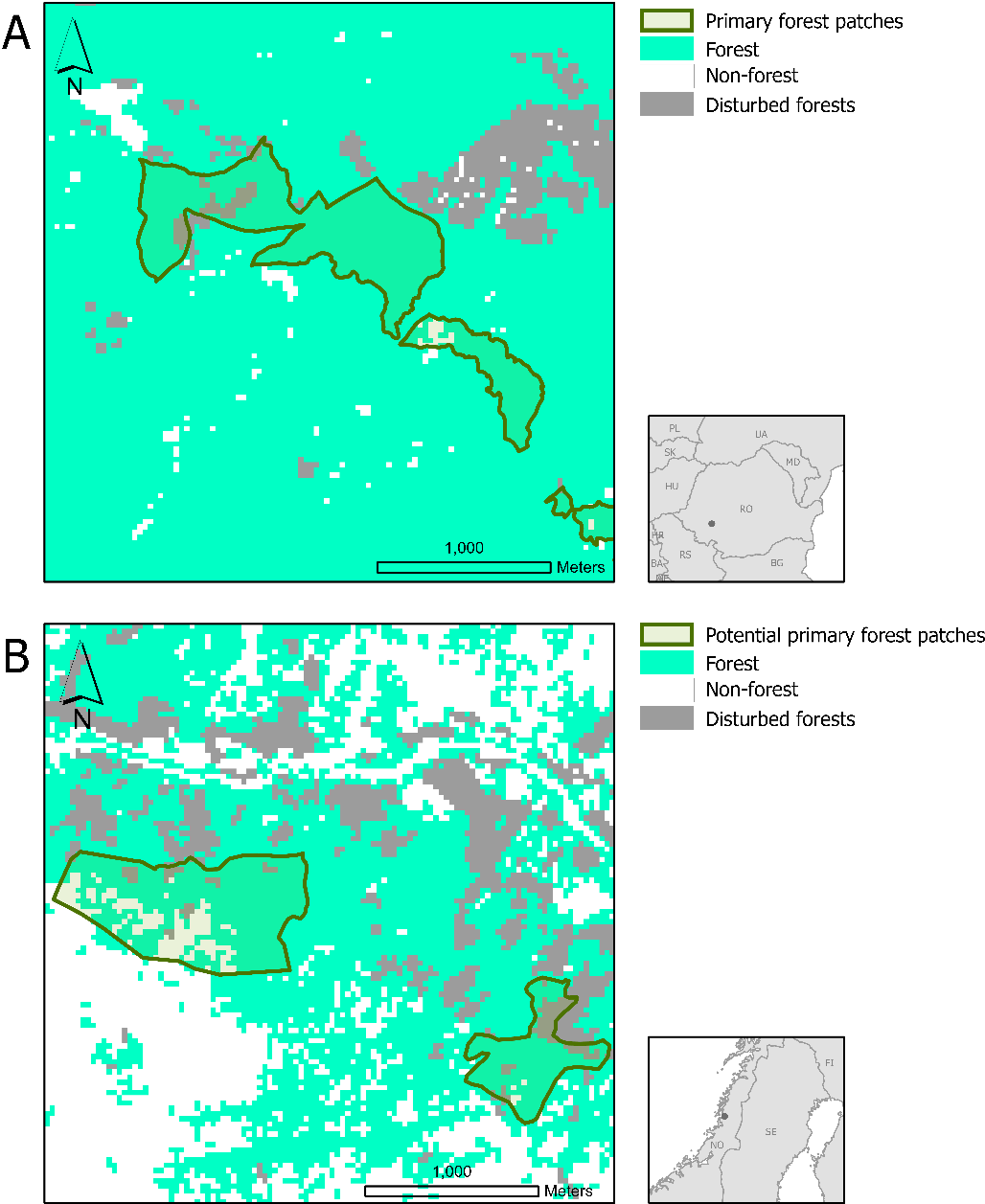


**Appendix S2**. Statistical analysis

The 95% confidence intervals (CIs) for the total area disturbed (1986–2020), the mean annual rate of area disturbed (1986–2020), and the mean patch disturbance severity (1986–2016) were calculated using a bootstrapping approach with replacement over 10,000 iterations (Davison & Hinkley, 1997; Efron, 1992; Efron & Tibshirani, 1994). For example, we used the series of annual area disturbed at the country level for bootstrapping. This process generated a distribution of 10,000 samples of the sum of the total area disturbed for each country. Subsequently, we calculated the 2.5th and 97.5th percentiles of the bootstrap distribution. The values at these percentiles correspond to the lower and upper bounds of the 95% confidence intervals, respectively. We applied the same method for calculating the CIs at the biogeographical, European, and EU levels, in this instance using data for these spatial units instead of country-specific data. We followed the same approach for calculating the CIs of both the mean annual rate of area disturbed and mean patch disturbance severity, utilizing the respective datasets.

We tested for a statistically significant difference in mean patch disturbance severity in primary forests before and after the mapping year. We separated the patches that occurred in the period predating the mapping year from those in the postdating period. Subsequently, for each country, we employed a non-parametric Mann-Whitney U test on samples from the predating and postdating periods, each consisting of a maximum of 1000 patches, selected to avoid the effects of spatial autocorrelation. In addition to countries, we conducted the test across the other three spatial scales: biogeographical region, Europe, and the EU.

To assess whether there was a statistically significant difference in the mean annual rates of area disturbed in primary forests predating and postdating the mapping year, we employed a non-parametric bootstrapping with replacement approach over 10,000 iterations (Davison & Hinkley, 1997; Efron & Tibshirani, 1994). We implemented this method in three steps. First, we calculated the mean annual rate of area disturbed in the predating and postdating periods across 64 spatial units that resulted from the combination of 40 country territories with 48 data sets of primary forests. This step addressed the variability in the mapping year across different data sets, which delineate the pre- and postdating periods. The mean annual rate of area disturbed, expressed as a percentage per year, was determined by dividing the total area disturbed in primary forests by the total area of primary forest and by the number of years in each period for every spatial unit.

Second, we calculated the mean annual rate of area disturbed for each country in the predating and postdating periods as the mean rate of the spatial units corresponding to each country. To control for the effect of differing primary forest areas within the spatial units, we calculate the mean annual rate for the countries by weighting the rate of each spatial unit with the primary forest area of that unit. This produced the mean annual rate of area disturbed per country for both the predating and postdating periods. With this, we then calculated the difference in the rates between the 2 periods for each country.

Third, we implemented a non-parametric bootstrapping with replacement method, conducting 10,000 iterations based on the annual data of area disturbed for each spatial unit. Consequently, we calculated a bootstrapped sample of the mean annual rate of area disturbance for each country for the predating and postdating periods, following the methodology outlined in the first and second steps. This process generated a distribution of 10,000 samples of the mean annual rate of area disturbed for each country for both periods, along with their differences. The resulting distribution, which approximates a normal distribution, was used to determine the *p*-value (Davison & Hinkley, 1997; Efron & Tibshirani, 1994). In this context, the *p*-value indicates the likelihood of observing a mean rate difference as extreme as, or more extreme than, the one actually observed mean rate difference, effectively conducting a two-tailed test, assessing the statistical significance of the difference in means rates.

**Appendix S3**. For primary forest (PF) in Europe, area, proportion of forest area, area disturbed, and mean annual rate of area disturbed predating (including mapping year) and postdating the primary forest’s mapping year by European countries from 1986 to 2020.

| **Country** | **Area (ha)** | **Proportion of total forest area (%)^a^** | **Area disturbed (ha) predating the mapping year (95% CI)** | **Mean rate of PF area disturbed (%/year) predating the mapping year (95% CI)** | **Area disturbed (ha) postdating the mapping year (95% CI)** | **Mean rate of PF area disturbed (%/year) postdating the mapping year (95% CI)** | **Change in mean rate of PF area disturbed (%/y)^b^** |
| --- | --- | --- | --- | --- | --- | --- | --- |
| Albania | 9660 | 0.9 | 329 (69−589) | 0.11 (0.02−0.19) | 36 (30−42) | 0.12 (0.09−0.14) | 0.01 |
| Andorra | 0 | − | − | − | − | − | − |
| Austria | 5769 | 0.1 | 147 (93−201) | 0.12 (0.04−0.20) | 123 (53−193) | 0.15 (0.06−0.24) | 0.03 |
| Belarus | 162,740 | 1.6 | 14,056 (8,525−19,587) | 0.27 (0.16−0.37) | 2,707 (1,361−4,053) | 0.55 (0.28−0.83) | 0.28 |
| Belgium | 262 | <0.1 | 2 (0−4) | 0.04 (0.01−0.08) | 2 (0−5) | 0.06 (0.00−0.15) | 0.02 |
| Bosnia and H. | 1672 | 0.1 | 4 (0−8) | 0.01 (0.00−0.02) | 0 | − | − |
| Bulgaria | 51,122 | 1.1 | 452 (321−583) | 0.03 (0.02−0.04) | 128 (77−179) | 0.04 (0.03−0.06) | 0.01 |
| Croatia | 8376 | 0.3 | 242 (155−329) | 0.11 (0.04−0.18) | 33 (14−52) | 0.05 (0.01−0.08) | -0.06* |
| Czech Republic | 7038 | 0.2 | 395 (297−493) | 0.18 (0.14−0.23) | 77 (43−111) | 0.27 (0.15−0.39) | 0.09 |
| Cyprus | 0 | − | − | − | − | − | − |
| Denmark | 0 | − | − | − | − | − | − |
| Estonia | 0 | − | − | − | − | − | − |
| Finland | 1,772,615 | 6.8 | 36,399 (29,922−42,876) | 0.07 (0.05−0.09) | 7,846 (4,674−11,018) | 0.10 (0.07−0.14) | 0.04* |
| France | 4993 | <0.1 | 62 (40−84) | 0.04 (0.02−0.07) | 7 (3−11) | 0.02 (0.00−0.04) | -0.02 |
| Germany | 5076 | <0.1 | 278 (185−371) | 0.18 (0.09−0.28) | 35 (21−49) | 0.12 (0.07−0.17) | -0.07 |
| Greece | 1563 | <0.1 | 16 (0−32) | 0.04 (0.00−0.07) | 2 (0−5) | 0.02 (0.00−0.04) | -0.02 |
| Hungary | 10 | <0.1 | 0 | − | 0 | − | − |
| Ireland | 0 | − | − | − | − | − | − |
| Italy | 7001 | 0.1 | 71 (38−104) | 0.03 (0.01−0.06) | 22 (9−35) | 0.04 (0.01−0.07) | 0.01 |
| Latvia | 4194 | 0.1 | 1163 (0−2549) | 0.84 (0.00−1.84) | 0 | − | − |
| Liechtenstein | 0 | − | − | − | − | − | − |
| Lithuania | 28,159 | 1.1 | 1041 (769−1313) | 0.12 (0.09−0.15) | 27 (16−38) | 0.02 (0.01−0.03) | -0.10** |
| Luxemburg | 0 | − | − | − | − | − | − |
| Malta | 0 | − | − | − | − | − | − |
| Moldova | 0 | − | − | − | − | − | − |
| Montenegro | 3186 | 0.4 | 40 (10−70) | 0.04 (0.00−0.11) | 5 (0−11) | 0.03 (0.00−0.07) | -0.01 |
| Netherlands | 79 | <0.1 | 0 | − | 0 | − | − |
| N. Macedonia | 719 | 0.1 | 17 (8−26) | 0.08 (0.03−0.13) | 0 | − | − |
| Norway | 139,294 | 1.2 | 2,840 (2,483−3,197) | 0.06 (0.05−0.08) | 418 (327−509) | 0.12 (0.09−0.15) | 0.06** |
| Poland | 19,900 | 0.2 | 314 (208−420) | 0.10 (0.07−0.14) | 1,861 (1,389−2,333) | 0.47 (0.35−0.59) | 0.36** |
| Portugal | 731 | <0.1 | 125 (80−170) | 0.55 (0.36−0.74) | 88 (0−188) | 3.02 (0.00−6.45) | 2.47** |
| Romania | 60,518 | 0.7 | 772 (606−938) | 0.04 (0.02−0.05) | 97 (49−145) | 0.05 (0.03−0.07) | 0.01 |
| Serbia | 869 | <0.1 | 1 (0−2) | 0.01 (0.00−0.03) | 6 (0−12) | 0.09 (0.00−0.20) | 0.09* |
| Slovakia | 11,918 | 0.5 | 777 (500−1,054) | 0.23 (0.14−0.32) | 186 (98−274) | 0.27 (0.12−0.43) | 0.05 |
| Slovenia | 8719 | 0.6 | 89 (39−139) | 0.03 (0.02−0.05) | 51 (32−70) | 0.12 (0.07−0.16) | 0.08** |
| Spain | 734 | <0.1 | 1 (0−2) | 0.01 (0.00−0.02) | 0 | − | − |
| Sweden | 6 | <0.1 | 0 | − | 0 | − | − |
| Switzerland | 506 | <0.1 | 7 (3−11) | 0.04 (0.02−0.07) | 1 (0−2) | 0.05 (0.00−0.13) | 0.01 |
| Ukraine | 83,097 | 0.6 | 2,194 (1,724−2,664) | 0.08 (0.06−0.10) | 629 (573−685) | 0.64 (0.62−0.66) | 0.56** |
| United Kingdom | 0 | − | − | − | − | − | − |
| Total Europe | 2,400,529 | 1.1 | 61,836 (53,402−70,270) | 0.08 (0.03−0.14) | 14,388 (11,781−16,995) | 0.15 (0.08−0.23) | 0.07** |
| Total EU | 1,998,785 | 1.1 | 42,347 (35,932−48,762) | 0.07 (0.02−0.12) | 10,585 (7,965−13,205) | 0.10 (0.05−0.16) | 0.04* |

^a^ According to the forest mask of Senf and Seidl (2021).

^b^ Mean rate of PF area disturbed postdating the mapping year minus mean rate of PF area disturbed predating the mapping year.

Significance: * *p* < 0.10; ** *p* < 0.05.

**Appendix S4.** Sensitivity Analysis

We implemented a sensitivity analysis to assess the potential impact of variations in the mapping year for primary forest data sets on the mean rate of area disturbed in each country, the EU, and at the European level. This analysis is useful, given that the mapping year associated with the primary forest data sets may be subject to some subjectivity due to factors such as the duration of the field survey, updates to the survey, and other considerations that may affect the assignment of the mapping year.

We introduced a systematic variation of ±1, ±2, and ±3 years to the mapping year for all primary forest data sets. Specifically, for each data set, we modified the mapping year by first adding 1, 2, and 3 years and then subtracting 1, 2, and 3 years. This created a testing range of 7 years (3 years before, the baseline year, and 3 years after), with the documented mapping year of each data set (baseline) at the centre. If the change in the mapping year resulted in a year beyond 2019, we capped the value at 2019 to ensure that each data set had at least one year in the period after the mapping year. Thus, the sensitivity analysis yielded six alternative scenarios in addition to the baseline case. We then calculated the difference in the mean rate of primary forest area disturbed between the postdating and predating periods for each country, the EU, and Europe for the six scenarios.

Results for the mean rate of primary forest area disturbed according to the six scenarios and the baseline for each country, the EU, and Europe are shown in the table in Appendix S5. A summary of this data is presented in the figure in Appendix S6. This figure includes only those countries that exhibited a mean rate of primary forest area disturbed in both the predating and postdating periods (see table in Appendix S3).

The sensitivity analysis results for Europe and the EU show that mean rate’s direction of change did not vary in any of the six scenarios (Appendix S6); the increase observed in the baseline is consistent across all scenarios. While the magnitude of change varies between the baseline and the scenarios, the trend towards an increase remains (see Appendix S5 for details). This pattern is also evident for the group of six countries with the majority of Europe's primary forests, where the mean rate consistently increases across all scenarios, despite great variability in magnitude compared to Europe and the EU. This consistency, particularly in the most extreme scenarios (subtracting and adding 3 years), underscores the robustness of our approach as it introduces substantial variation to the mapping year across all data sets. In other countries, like Lithuania and Poland, the scenario trends align with the baseline. However, in a few countries, such as Slovakia and Austria, there is variation in both magnitude and direction across some scenarios, indicating that changes in the mapping year may introduce uncertainty in these results. Yet, in 87% of the 150 cases, there is consistency in the direction between the baseline and the six scenarios, as depicted by the coloured circles in the figure in Appendix S6.

In summary, the sensitivity analysis suggested that potential uncertainties surrounding the mapping year of the primary forest data sets are unlikely to significantly alter the outcomes of this study within the assessed range of years. The observed increase in the mean annual rate of area disturbed between the predating and postdating period remains consistent even after introducing simulated changes to the mapping year of the primary forest data sets.

**Appendix S5.** Sensitivity analysis: mean rate of primary forest (PF) area disturbed. Baseline represents the rate using the mapping year documented in the PF data sets. *Subtracting* *n years* shows the rate by subtracting *n* years to the mapping year of the PF data sets. *Adding n years* shows the rate by adding *n* years to the mapping year of the PF data sets.

|  |  | **Mean rate of primary forest area disturbed predating and postdating the mapping year (%/y)** | | | | | | | | | | | | | |
| --- | --- | --- | --- | --- | --- | --- | --- | --- | --- | --- | --- | --- | --- | --- | --- |
|  |  | **Baseline** | | **Subtracting 3 years** | | **Subtracting 2 years** | | **Subtracting 1 year** | | **Adding 1 year** | | **Adding 2 years** | | **Adding 3 years** | |
| **Country** | **Area of PF (ha)** | **Predating** | **Postdating** | **Predating** | **Postdating** | **Predating** | **Postdating** | **Predating** | **Postdating** | **Predating** | **Postdating** | **Predating** | **Postdating** | **Predating** | **Postdating** |
| Albania | 9660 | 0.11 | 0.12 | 0.11 | 0.08 | 0.11 | 0.08 | 0.11 | 0.10 | 0.10 | 0.21 | 0.10 | 0.21 | 0.10 | 0.21 |
| Austria | 5769 | 0.12 | 0.15 | 0.10 | 0.16 | 0.10 | 0.17 | 0.10 | 0.17 | 0.13 | 0.14 | 0.14 | 0.11 | 0.14 | 0.09 |
| Belarus | 162,740 | 0.27 | 0.55 | 0.20 | 0.73 | 0.23 | 0.66 | 0.27 | 0.52 | 0.27 | 0.72 | 0.29 | 0.31 | 0.29 | 0.31 |
| Belgium | 262 | 0.04 | 0.06 | 0.05 | 0.05 | 0.05 | 0.05 | 0.05 | 0.06 | 0.04 | 0.07 | 0.04 | 0.07 | 0.04 | 0.08 |
| Bosnia and H. | 1672 | 0.01 | − | 0.01 | − | 0.01 | − | 0.01 | − | 0.01 | − | 0.01 | − | 0.01 | − |
| Bulgaria | 51,122 | 0.03 | 0.04 | 0.03 | 0.04 | 0.03 | 0.04 | 0.03 | 0.04 | 0.03 | 0.05 | 0.03 | 0.07 | 0.03 | 0.08 |
| Croatia | 8376 | 0.11 | 0.05 | 0.10 | 0.09 | 0.10 | 0.09 | 0.10 | 0.08 | 0.11 | 0.03 | 0.11 | 0.02 | 0.11 | 0.02 |
| Czech Republic | 7038 | 0.18 | 0.27 | 0.19 | 0.18 | 0.19 | 0.20 | 0.19 | 0.22 | 0.18 | 0.34 | 0.18 | 0.37 | 0.19 | 0.42 |
| Finland | 1,772,615 | 0.07 | 0.10 | 0.07 | 0.08 | 0.07 | 0.09 | 0.07 | 0.09 | 0.07 | 0.12 | 0.07 | 0.12 | 0.07 | 0.12 |
| France | 4993 | 0.04 | 0.02 | 0.05 | 0.02 | 0.05 | 0.02 | 0.04 | 0.02 | 0.04 | 0.02 | 0.04 | 0.01 | 0.04 | 0.01 |
| Germany | 5076 | 0.18 | 0.12 | 0.20 | 0.08 | 0.20 | 0.08 | 0.19 | 0.09 | 0.18 | 0.13 | 0.18 | 0.08 | 0.18 | 0.10 |
| Greece | 1563 | 0.04 | 0.02 | 0.04 | 0.03 | 0.03 | 0.03 | 0.03 | 0.03 | 0.04 | 0.02 | 0.03 | 0.02 | 0.03 | 0.03 |
| Italy | 7001 | 0.03 | 0.04 | 0.04 | 0.03 | 0.03 | 0.03 | 0.03 | 0.04 | 0.03 | 0.05 | 0.03 | 0.05 | 0.03 | 0.05 |
| Latvia | 4194 | 0.84 | − | 0.92 | − | 0.89 | − | 0.87 | − | 0.82 | − | 0.82 | − | 0.82 | − |
| Lithuania | 28,159 | 0.12 | 0.02 | 0.12 | 0.04 | 0.13 | 0.03 | 0.12 | 0.03 | 0.12 | 0.03 | 0.11 | 0.03 | 0.11 | 0.04 |
| Montenegro | 3186 | 0.04 | 0.03 | 0.04 | 0.03 | 0.04 | 0.02 | 0.04 | 0.02 | 0.04 | 0.04 | 0.04 | 0.04 | 0.04 | 0.06 |
| North Macedonia | 719 | 0.08 | − | 0.09 | − | 0.08 | − | 0.08 | − | 0.08 | − | 0.08 | − | 0.07 | − |
| Norway | 139,294 | 0.06 | 0.12 | 0.06 | 0.10 | 0.06 | 0.11 | 0.06 | 0.16 | 0.06 | 0.26 | 0.06 | 0.27 | 0.06 | 0.30 |
| Poland | 19,900 | 0.10 | 0.47 | 0.10 | 0.42 | 0.10 | 0.44 | 0.10 | 0.45 | 0.10 | 0.49 | 0.11 | 0.50 | 0.12 | 0.52 |
| Portugal | 731 | 0.55 | 3.02 | 0.49 | 2.20 | 0.50 | 2.44 | 0.54 | 2.60 | 0.56 | 3.79 | 0.83 | 0.93 | 0.85 | 0.22 |
| Romania | 60,518 | 0.04 | 0.05 | 0.04 | 0.06 | 0.04 | 0.07 | 0.04 | 0.05 | 0.04 | 0.07 | 0.04 | 0.07 | 0.04 | 0.07 |
| Serbia | 869 | 0.01 | 0.09 | 0.01 | 0.07 | 0.01 | 0.08 | 0.01 | 0.09 | 0.01 | 0.08 | 0.02 | 0.09 | 0.04 | 0.01 |
| Slovakia | 11,918 | 0.23 | 0.27 | 0.23 | 0.27 | 0.22 | 0.28 | 0.24 | 0.23 | 0.22 | 0.32 | 0.24 | 0.18 | 0.24 | 0.17 |
| Slovenia | 8719 | 0.03 | 0.12 | 0.02 | 0.13 | 0.02 | 0.14 | 0.03 | 0.11 | 0.03 | 0.14 | 0.04 | 0.15 | 0.04 | 0.14 |
| Spain | 734 | 0.01 | − | 0.01 | − | 0.01 | − | 0.01 | − | 0.01 | − | 0.01 | − | 0.01 | − |
| Switzerland | 506 | 0.04 | 0.05 | 0.04 | 0.06 | 0.04 | 0.05 | 0.05 | 0.04 | 0.05 | 0.01 | 0.05 | 0.01 | 0.05 | 0.00 |
| Ukraine | 83,097 | 0.08 | 0.64 | 0.07 | 0.30 | 0.08 | 0.32 | 0.08 | 0.39 | 0.08 | 0.64 | 0.08 | 0.64 | 0.08 | 0.64 |
| Total Europe | 2,400,529 | 0.08 | 0.15 | 0.08 | 0.14 | 0.08 | 0.13 | 0.08 | 0.12 | 0.08 | 0.13 | 0.08 | 0.14 | 0.08 | 0.16 |
| Total EU | 1,998,785 | 0.07 | 0.10 | 0.07 | 0.09 | 0.07 | 0.08 | 0.07 | 0.08 | 0.07 | 0.08 | 0.07 | 0.09 | 0.07 | 0.10 |

**Appendix S6.** Sensitivity analysis: Extent of primary forests (left panel) and difference (change) between the mean rate of primary forest area disturbed in the postdating and predating periods of the primary forest data sets by country, Europe and, the EU (right panel). Baseline corresponds to the rate calculated using the mapping year documented in the primary forest data sets. *Subtracting n years* corresponds to the rate calculated for the scenarios subtracting *n* years to the mapping year of the primary forest data sets. *Adding n years* corresponds to the rate calculated for the scenarios adding *n* years to the mapping year of the primary forest data sets. First graph is in log scale. Vertical gray line, zero or no change.

**
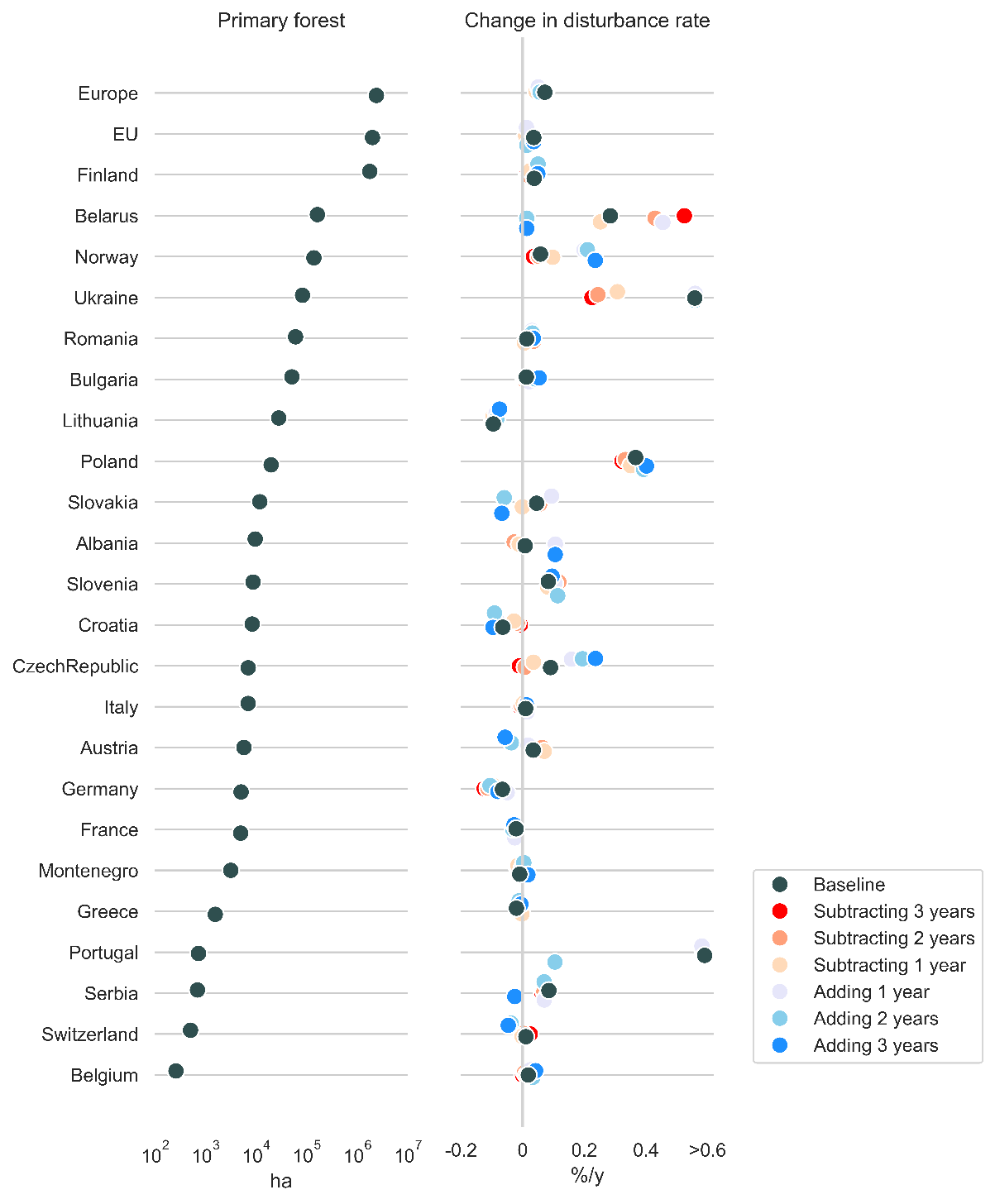
**

**Appendix S7**. For primary forest (PF) in Europe, mean patch disturbance severity (range: 0−1) predating (including mapping year) and postdating the primary forest’s mapping year by European countries from 1986 to 2016.

| **Country** | **Mean patch disturbance severity predating the mapping year (95% CI)** | **Mean patch disturbance severity postdating the mapping year (95% CI)** | **Change in mean patch disturbance severity between the predating and postdating mapping year**^a^ |
| --- | --- | --- | --- |
| Albania | 0.47 (0.45−0.48) | 0.31 (0.25−0.36) | -0.16* |
| Andorra | − | − | − |
| Austria | 0.61 (0.59−0.63) | 0.56 (0.54−0.59) | -0.05* |
| Belarus | 0.66 (0.66−0.66) | 0.69 (0.68−0.70) | 0.03* |
| Belgium | 0.72 (0.57−0.88) | 0.80 (0.74−0.88) | 0.07 |
| Bosnia and Herzegovina | 0.65 (0.51−0.78) | − | − |
| Bulgaria | 0.54 (0.52−0.55) | 0.53 (0.50−0.56) | -0.01 |
| Croatia | 0.51 (0.49−0.53) | 0.53 (0.49−0.57) | 0.02 |
| Czech Republic | 0.67 (0.65−0.69) | 0.56 (0.52−0.60) | -0.11* |
| Cyprus | − | − | − |
| Denmark | − | − | − |
| Estonia | − | − | − |
| Finland | 0.693 (0.69−0.70) | 0.692 (0.69−0.69) | -0.001* |
| France | 0.72 (0.68−0.76) | 0.76 (0.66−0.86) | 0.05 |
| Germany | 0.66 (0.64−0.67) | 0.55 (0.48−0.62) | -0.11* |
| Greece | 0.52 (0.39−0.64) | 0.72 (0.70−0.73) | 0.20 |
| Hungary | − | − | − |
| Ireland | − | − | − |
| Italy | 0.61 (0.57−0.65) | 0.51 (0.45−0.58) | -0.10* |
| Latvia | 0.58 (0.57−0.59) | − | − |
| Liechtenstein | − | − | − |
| Lithuania | 0.62 (0.61−0.63) | 0.72 (0.66−0.78) | 0.10* |
| Luxemburg | − | − | − |
| Malta | − | − | − |
| Moldova | − | − | − |
| Montenegro | 0.62 (0.57−0.66) | 0.50 (0.42−0.56) | -0.12* |
| Netherlands | − | − | − |
| North Macedonia | 0.59 (0.54−0.64) | − | − |
| Norway | 0.70 (0.69−0.70) | 0.72 (0.71−0.73) | 0.02* |
| Poland | 0.67 (0.65−0.69) | 0.66 (0.65−0.66) | -0.01* |
| Portugal | 0.67 (0.65−0.70) | 0.74 (0.67−0.81) | 0.07* |
| Romania | 0.53 (0.51−0.54) | 0.39 (0.36−0.42) | -0.14* |
| Serbia | 0.61 (0.34−0.78) | 0.70 (0.50−0.86) | 0.10 |
| Slovakia | 0.66 (0.65−0.67) | 0.57 (0.55−0.59) | -0.09* |
| Slovenia | 0.34 (0.31−0.37) | 0.51 (0.46−0.55) | 0.17* |
| Spain | 0.51 (0.23−0.79) | − | − |
| Sweden | − | − | − |
| Switzerland | 0.60 (0.52−0.69) | 0.74 (0.59−0.90) | 0.14 |
| Ukraine | 0.69 (0.69−0.70) | 0.66 (0.65−0.68) | -0.03* |
| United Kingdom | − | − | − |
| Total Europe | 0.67 (0.67−0.68) | 0.68 (0.68−0.68) | 0.0046 |
| Total EU | 0.68 (0.68−0.68) | 0.68 (0.68−0.68) | -0.0002 |

^a^ Mean patch disturbance severity postdating the mapping year minus mean patch disturbance severity predating the mapping year. Significance: * *p* < 0.05.

**Appendix S8**. For potential primary forest (PPF) in Europe, area, proportion of total forest area, area disturbed, mean annual rate of area disturbed, and mean disturbance severity (range: 0−1) by European countries from 1986 to 2020.

| **Country** | **Area (ha)** | **Proportion of total forest area (%)^a^** | **Area disturbed (ha) 1986−2020 (95% CI)** | | **Proportion of PPF area disturbed**  **1986-2020 (%)** | **Mean rate (%/y) of PPF area disturbed 1986-2020 (95% CI)** | **Mean severity (range 0-1)**  **(95% CI)^b^** |
| --- | --- | --- | --- | --- | --- | --- | --- |
| Albania | 0 | − | − | | − | − | − |
| Andorra | 0 | − | − | | − | − | − |
| Austria | 0 | − | − | | − | − | − |
| Belarus | 0 | − | − | | − | − | − |
| Belgium | 0 | − | − | | − | − | − |
| Bosnia and H. | 0 | − | − | | − | − | − |
| Bulgaria | 98,236 | 2.1 | 4,027 (3,351−4,804) | | 4.1 | 0.12 (0.10−0.14) | 0.57 (0.57−0.58) |
| Croatia | 0 | − | − | | − | − | − |
| Czech Republic | 0 | − | − | | − | − | − |
| Cyprus | 0 | − | − | | − | − | − |
| Denmark | 0 | − | − | | − | − | − |
| Estonia | 0 | − | − | | − | − | − |
| Finland | 0 | − | − | | − | − | − |
| France | 0 | − | − | | − | − | − |
| Germany | 0 | − | − | | − | − | − |
| Greece | 125 | <0.1 | 1 (0−3) | | 0.8 | 0.02 (0.00−0.07) | 0.56 (0.29−0.80) |
| Hungary | 0 | − | − | | − | − | − |
| Ireland | 0 | − | − | | − | − | − |
| Italy | 0 | − | − | | − | − | − |
| Latvia | 0 | − | − | | − | − | − |
| Liechtenstein | 0 | − | − | | − | − | − |
| Lithuania | 0 | − | − | | − | − | − |
| Luxemburg | 0 | − | − | | − | − | − |
| Malta | 0 | − | − | | − | − | − |
| Moldova | 0 | − | − | | − | − | − |
| Montenegro | 0 | − | − | | − | − | − |
| Netherlands | 0 | − | − | | − | − | − |
| North Macedonia | 14 | <0.1 | 2 (0−3) | | 11.1 | 0.32 (0.00−0.62) | 0.67 (0.51−0.83) |
| Norway | 86,566 | 0.7 | 3,324 (2,786−3,984) | | 3.8 | 0.11 (0.09−0.13) | 0.72 (0.71−0.72) |
| Poland | 0 | − | − | | − | − | − |
| Portugal | 0 | − | − | | − | − | − |
| Romania | 593,832 | 6.8 | 11,250 (6,803−18,198) | | 1.9 | 0.05 (0.03−0.09) | 0.50 (0.49−0.50) |
| Serbia | 64 | <0.1 | 1 (0−3) | | 1.8 | 0.05 (0.00−0.13) | 0.49 (0.20−0.78) |
| Slovakia | 0 | − | − | | − | − | − |
| Slovenia | 0 | − | − | | − | − | − |
| Spain | 0 | − | − | | − | − | − |
| Sweden | 2,164,483 | 6.8 | 42,397 (29,125−59,484) | | 2.0 | 0.06 (0.04−0.08) | 0.67 (0.67−0.67) |
| Switzerland | 0 | − | − |  | − | − | − |
| Ukraine | 7 | <0.1 | 0 | | − | − | − |
| United Kingdom | 0 | − | − | | − | − | − |
| Total Europe | 2,943,327 | 1.3 | 61,003 (44,905−80,408) | | 2.1 | 0.06 (0.04−0.08) | 0.64 (0.63−0.64) |
| Total EU | 2,856,676 | 1.6 | 57,675 (41,915−76,677) | | 2.0 | 0.06 (0.04−0.08) | 0.63 [0.63−0.63) |

^a^ According to the forest mask of Senf and Seidl (2021).

^b^ Period 1986-2016.

**Appendix S9.** Comparison between the combined mapped area of primary and potential primary forests and ancillary sources

The comparison between the combined area of primary and potential primary forests calculated in this study and that reported by the FRA reveals that 7 out of 40 countries provided no data in either this study or the FRA: Andorra, Cyprus, Ireland, Luxembourg, Malta, Moldova, and UK. Furthermore, 10 countries reported data in this study but not in FRA: Belgium, Bosnia and Herzegovina, France, Germany, Greece, Hungary, Netherlands, North Macedonia, Poland, and Spain. Conversely, 3 countries reported data in the FRA, but not in this study, specifically Denmark (21,140 ha), Estonia (52,090 ha), and Liechtenstein (2000 ha). The latter discrepancy suggests that there are gaps in the maps of primary and potential primary forest used in this study.

In the remaining 20 countries, the proportion between the estimated area in this study and the area of primary forests reported by the FRA at the country-level exhibits pronounced variability. The area mapped in this study was greater than that reported in the FRA in 9 countries; these included Lithuania, Ukraine and Belarus in Eastern Europe, as well as Croatia, Finland, Romania, Slovakia, Montenegro, and Norway in Western Europe. Conversely, our estimation was lower in 11 countries, namely Austria, Bulgaria, Czechia, Italy, Latvia, Portugal, Slovenia, Sweden, Albania, Serbia, and Switzerland. Collectively, the estimated shortfall in these countries exceeds 964,000 ha. The majority of this discrepancy is observed in Bulgaria, which reported 704,000 ha to the FRA, while our mapping accounted for only 149,358 ha, indicating a gap of 554,642 ha. Pronounced gaps were also observed in Italy (85,999 ha), Sweden (84,510 ha), Albania (75,340), and Austria (57,231 ha).

In the 4 countries that host nearly all of the potential primary forests, we compared the area documented in this study with information from additional sources (Appendix S10). The results of the comparison show that in Sweden, the total combined area of 2.16 million ha is 7.4% greater than the nearly 2 million ha reported by Andersson (2021) and is 4.2% lower than the 2.25 million ha of primary forests reported in the FRA. Romania accounted for a combined area of 0.65 million ha, which is 13.8% lower than the 0.74 million ha of high-conservation-value forests (HCVF) mapped in Munteanu et al. (2022). The definition of HCVF partially overlaps with the definition of primary forests adopted in this study. However, more than half of the HCVF are subject to anthropogenic pressures, meaning that only around 351,000 ha of HCVF resemble primary forests (Munteanu et al., 2022). This could imply an overestimation of our mapped area in Romania. Similarly, the area reported by the FRA for Romania is considerably lower than both the combined area in this study and that reported by Munteanu et al. (2022), suggesting a possible overestimation in our combined area based on the maps used in this study compared with the FRA. However, whether forests in the overestimated area can possess old-growth attributes, which are key for conservation, warrants further investigation.

The total combined area considered in Norway amounts to 0.23 million ha, which exceeds the 0.2 million ha reported in the FRA by 13%. No further ancillary information was found for Norway. In Bulgaria, the combined area of primary and potential primary forests is 0.15 million ha. This figure is one-third higher than the 0.1 million ha of virgin forests mapped in Veen et al. (2010), although the definition of virgin forests is more restrictive. Notably, Bulgaria reported 0.7 million ha of primary forests to the FRA, indicating a substantial data gap for the area mapped for this country in this study.

**Appendix S10**. Comparison of the combined area of primary and potential primary forests in this study with information on primary forest and high-conservation-value forest (HCVF) from other sources in the 4 countries with the most potential primary forests in Europe.

| **Country** | **Combined primary and potential primary forests (this study) (million ha)** | **Primary forest**  **(million ha) (source)** | **Primary forest - Global Forest Resources Assessment**  **(million ha) (FAO, 2020)** |
| --- | --- | --- | --- |
| Sweden | 2.16 | 2.0  (Andersson, 2021) | 2.25 |
| Romania | 0.65 | 0.74 (HCVF)  (Munteanu et al., 2022) | 0.17 |
| Norway | 0.23 | − | 0.2 |
| Bulgaria | 0.15 | 0.1  (Veen et al., 2010) | 0.7 |

**References**

Andersson, J. (2021). *Forestry at the Edge - Results from the 2020 forest inventory of large forest landscapes*. Protect the Forest.

Davison, A. C., & Hinkley, D. V. (1997). *Bootstrap Methods and their Application*. Cambridge University Press. https://doi.org/10.1017/CBO9780511802843

Efron, B. (1992). Bootstrap Methods: Another Look at the Jackknife. In S. Kotz & N. L. Johnson (Eds.), *Breakthroughs in Statistics: Methodology and Distribution* (pp. 569-593). Springer New York. <https://doi.org/10.1007/978-1-4612-4380-9_41>

Efron, B., & Tibshirani, R. J. (1994). *An Introduction to the Bootstrap*. Chapman and Hall/CRC. <https://doi.org/10.1201/9780429246593>

FAO. (2020). *Global Forest Resources Assessment 2020: Main Report*. Food and Agriculture Organization of the United Nations.

Munteanu, C., Senf, C., Nita, M. D., Sabatini, F. M., Oeser, J., Seidl, R., & Kuemmerle, T. (2022). Using historical spy satellite photographs and recent remote sensing data to identify high-conservation-value forests. *Conservation Biology*, *36*, e13820.

Sabatini, F. M., Bluhm, H., Kun, Z., Aksenov, D., Atauri, J. A., Buchwald, E., Burrascano, S., Cateau, E., Diku, A., Duarte, I. M., Fernández López, Á. B., Garbarino, M., Grigoriadis, N., Horváth, F., Keren, S., Kitenberga, M., Kiš, A., Kraut, A., Ibisch, P. L., . . . Kuemmerle, T. (2021a). European primary forest database v2.0. *Scientific Data*, *8*(1), 220.

Sabatini, F. M., Bluhm, H., Kun, Z., Aksenov, D., Atauri, J. A., Buchwald, E., Burrascano, S., Cateau, E., Diku, A., Duarte, I. M., López, Á. B. F., Garbarino, M., Grigoriadis, N., Horváth, F., Keren, S., Kitenberga, M., Kiš, A., Kraut, A., Ibisch, P. L., . . . Kuemmerle, T. (2021b). *European Primary Forest Database.* Version 2.0. Figshare. <https://doi.org/10.6084/m9.figshare.13194095.v2>

Senf, C., & Seidl, R. (2020). *European forest disturbance map (1.0.0) [Data set]. Zenodo.* <https://doi.org/10.5281/zenodo.3924381>

Senf, C., & Seidl, R. (2021). Mapping the forest disturbance regimes of Europe. *Nature Sustainability*, *4*(1), 63-70.

Veen, P., Fanta, J., Raev, I., Biriş, I.-A., de Smidt, J., & Maes, B. (2010). Virgin forests in Romania and Bulgaria: results of two national inventory projects and their implications for protection. *Biodiversity and Conservation*, *19*(6), 1805-1819.
